# Systematic review and meta-analysis of homicide recidivism and Schizophrenia

**DOI:** 10.1186/1471-244X-14-46

**Published:** 2014-02-18

**Authors:** Andrei Golenkov, Olav Nielssen, Matthew Large

**Affiliations:** 1 Psychiatry and Medical Psychology, Chuvash State University, Cheboksary, Russia; 2 University of New South Wales, Sydney, Australia; 3 Clinical Research Unit for Anxiety and Depression, St Vincent’s Hospital, Sydney, Australia; 4 Prince of Wales Hospital, Barker St, Randwick, 2031 Sydney, NSW Australia

**Keywords:** Homicide, Schizophrenia, Psychosis, Recidivism, Repeat homicide, Violence

## Abstract

**Background:**

The aim of this study was to estimate the proportion of homicide recidivists among population studies of homicide offenders with schizophrenia.

**Methods:**

Systematic review and meta-analysis of published studies of homicide associated with schizophrenia conducted in defined populations and indexed in Medline, PsychINFO, or Embase between January 1960 and November 2013. Published data was supplemented with unpublished data about recidivism obtained by personal communication from the authors of published studies of homicide and schizophrenia. Random effects meta-analysis was used to calculate a pooled estimate of the proportion of homicide recidivists.

**Results:**

Three studies reported that 4.3%, 4.5%, and 10.7% of homicide offenders with schizophrenia had committed an earlier homicide. Unpublished data were obtained from the authors of 11 studies of homicide in schizophrenia published in English between 1980 and 2013. The authors of 2 studies reported a single case of homicide recidivism and the authors of 9 studies reported no cases. The rates of homicide recidivism between studies were highly heterogeneous (I-square = 79). The pooled estimate of the proportion of homicide offenders with schizophrenia who had committed an earlier homicide was 2.3% (95% CI (Confidence Interval) 0.07% to 7.2%), a figure that was not reported in any individual study. The pooled proportion of homicide recidivists from published reports was more than ten times greater (8.6%, 95% CI 5.7%-12.9%) than the pooled proportion of homicide recidivists estimated from data provided by personal communication (0.06%, 95% CI 0.02% to 1.8%).

**Conclusions:**

In most jurisdictions, homicide recidivism by people with schizophrenia is less common than published reports have suggested. The reasons for the variation in the rates of homicide recidivism between studies are unclear, although in most jurisdictions long-term secure treatment and supervision after release appears to be effective in preventing homicide recidivism. A prospective study conducted in a large population or in multiple jurisdictions over a long period of time might result in a more accurate estimate the risk of a second homicide by a person with schizophrenia.

## Background

Recidivist homicide has been defined as a homicide committed after the conclusion of proceedings for an earlier homicide offence [1]. Most jurisdictions have systems to determine when homicide offenders who are found to be not guilty by reason of insanity can be released to the community [2-6]. However, little is known about the probability of mentally ill offenders committing further homicides. Bjøkly and Waage conducted a systematic review of recidivism among homicide offenders and found a total of 6 studies from the USA, the UK, Sweden, Finland and Australia [1]. Of the studies included in their review, only a study from Finland provided any information about psychiatric diagnoses of the homicide recidivists. The authors of the Finnish study concluded that schizophrenia might be a risk factor for homicide recidivism because 4 (4.3%) of 93 homicide offenders with a diagnosis of schizophrenia were recidivists compared with 32 (2.1%) of 1499 other homicide offenders [7-10]. Another Nordic study from two regions in Sweden found that 1 (4.5%) of 22 homicide offenders with psychosis had committed an earlier homicide, and none committed a further homicide during a follow up period of 32 years [11]. A recent study from the Chuvash Republic of the Russian Federation, found that 16 (10.7%) of 149 offenders with schizophrenia had committed an earlier homicide [12]. However, most studies of homicide by people diagnosed with schizophrenia do not mention recidivist offenders [13-21]. Reports of homicide recidivism might be subject to publication bias if studies with more recidivists are more likely to be published, and the absence of reported cases of recidivism in most of the published series of homicide offenders with psychosis suggests that homicide recidivism in schizophrenia is rare.

Homicide recidivism by patients with schizophrenia is of interest because the data from Finland and Russia suggests that schizophrenia might be a potentially modifiable risk factor for homicide recidivism, and because most homicide offenders with schizophrenia are eventually released into the community. The aim of this study was to conduct a systematic review and meta-analysis of the available data about the proportion of recidivist homicide offenders with a diagnosis of schizophrenia in population based samples of homicide offenders with schizophrenia.

## Method

The methods used were based on the ‘MOOSE’ guidelines for conducting systematic reviews in epidemiology [22].

### Searches

**Figure 1:**
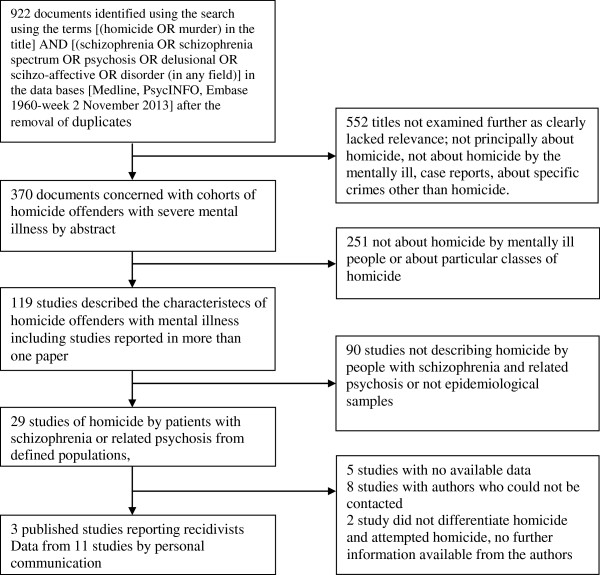
Flow chart of searches for published and unreported cases of homicide recidivism associated with schizophrenia.

A systematic review of the literature was conducted to locate publications in English describing epidemiological samples of homicide offenders with schizophrenia indexed in Medline, PsychINFO, or Embase between January 1960 and November, 2013. Epidemiological samples were defined as those including data on all the known homicide offenders in a defined geographic region over a specified period of time. The searches were supplemented by studies identified in a systematic meta-analysis of rates of homicide by offenders with schizophrenia [23] and a recent review of criminal recidivism of homicide offenders [24]. All papers that were likely to meet inclusion criteria were examined in full text and their references were hand searched for further studies (Figure 1). We excluded studies of the populations in special hospitals because of the likelihood that sampling bias would result in an overestimate of the probability of homicide recidivism.

### Inclusion and exclusion criteria

Studies were included if they:

•reported on homicides offenders from a defined geographic region over a specified period of time; and

•reported on homicide offenders with a diagnosis of schizophrenia or related psychosis including delusional disorder and schizo-affective disorder.

Studies were excluded if they:

•reported on samples of offenders who might not have been representative of all offenders in the population, such as those selected on the basis of incarceration in a special hospital; or

•did not differentiate homicide from attempted homicide; or

•used a definition of mental illness other than a diagnosis of schizophrenia or related psychosis according to a recognized psychiatric classification system (ICD or DSM).

### Data extraction and unpublished data

All of the studies that met the inclusion criteria were carefully examined for reports of people who had committed more than one homicide. Recidivist homicide was defined as a homicide committed after the conclusion of proceedings following a previous homicide. This definition excluded people who had committed a homicide and an attempted homicide, multiple homicides committed in a single incident, and spree or serial killings that took place before the offender was apprehended by the police [1].

The authors of published studies of homicide by people with schizophrenia that did not report cases of recidivism were contacted by email and asked if any of the offenders included in their studies had committed a previous homicide. All of the authors who provided data did so on the understanding that it was for the purpose of publication in a systematic review and meta-analysis. In order to allow valid comparisons with published data we limited our enquiries to data relating to homicides committed prior to the offences that resulted in inclusion in the published studies, rather than subsequent homicides. Each of the authors was asked an identical question that clearly expressed our interest in earlier homicides by patients included in their published series. The authors of studies that reported homicide and attempted homicide together were asked for separate data for the two types of offences.

Non-age-corrected total homicide rates per 100,000 per annum were reported directly in the majority of studies, or were calculated using official statistics for population at the midpoint of the study.

Two authors independently extracted the data from published studies with no discrepancies.

### Statistical analysis

Between-study heterogeneity was assessed using Q-value and I-square statistics and a *X*^2^ contingency table. A sensitivity analysis compared rates of homicide recidivism in published studies that reported homicide recidivism to the rates in studies where data about homicide recidivism was obtained by personal communication. A random effects model meta-analysis was used to calculate a pooled proportion of recidivists among offenders with schizophrenia using Comprehensive Meta Analysis (CMA) version 2.2 (Biostat, Engelwood, NJ, USA). All significance tests were conducted in a two-tailed form.

## Results

We located 29 studies reporting the characteristics of homicide offenders with schizophrenia from defined populations, including the studies located in an earlier meta-analysis of rates of homicide in schizophrenia [23] and 8 more recent papers [11,12,19,21,25-28]. Three published accounts of homicide recidivism that met our definition of an earlier homicide by a homicide offender with schizophrenia were from Finland [7], Sweden [11] and the Chuvash Republic of the Russian Federation [12,29]. A study from Finland that used a sample of homicide offenders that overlapped with an earlier study [7] reported that 10 (8%) of 125 homicide offenders with schizophrenia had committed either a homicide or an attempted homicide [16]. Two German studies of offenders with schizophrenia who had committed a homicide or an attempted homicide reported that 11 (3.9%) of 284 [30] and 4 (13.8%) of 29 offenders had committed an earlier homicide [31].

First, we contacted the authors the three studies that had reported homicide offences in schizophrenia defined more broadly by including attempted homicide [16,30,31]. One author was able to report that of none of the 17 offenders who had committed an actual homicide had committed an earlier homicide [31]. No further information about the number of cases that met our definition of homicide recidivism was available from the other two studies that reported a mix of homicide and attempted homicide offenders [16,30].

Second, we attempted to contact all of the authors of epidemiological studies of homicide in schizophrenia that had not mentioned the presence or absence of offenders with a history of a previous homicide offence. We were able to contact by email all but one [19] of the authors of epidemiological studies of homicide in schizophrenia published after 1981, but we were only able to contact the authors of one of the 8 studies published between published between 1960 and 1981. The authors of two studies were not able to provide further data because homicide recidivism was not part of their research design [27,32]. The authors of 10 studies provided previously unreported data about homicide recidivism. There was one case from New South Wales, Australia [6,13], and one case in a recent study from Saudi Arabia [26]. The authors of 8 other published studies from Austria [33], Australia (two studies from Victoria) [21,34], Barbados [35], New Zealand [36], The Netherlands [28], Nigeria [37] and Singapore [38] confirmed that there were no cases of recidivism in their samples. The authors of 3 studies from the UK confirmed the presence of some recidivists in their samples but they could not specify the number of cases [14,17,25]. Hence, of 29 studies reporting homicide offenders with schizophrenia from defined geographic regions, 3 studies reported homicide recidivism according to our definition and 3 studies reported broader definitions of homicide recidivism involving attempted homicide (1 of which provided further data that met our inclusion criteria). The authors of 10 studies that did not mention homicide recidivism provided previously unpublished data about homicide recidivism, the authors of 5 studies were unable to provide relevant data and the authors of 8 studies could not be contacted. In total we located data on 23 cases of homicide recidivism in schizophrenia among 801 homicide offenders with schizophrenia (2.9%) described in 14 studies conducted in 13 jurisdictions from 12 countries. There were no known cases of recidivism in 9 jurisdictions, 4 cases from Finland, 16 from the Chuvash Republic of the Russian Federation and one each from New South Wales, Australia, Saudi Arabia and Sweden (See Table 1).

**Table 1:** Published and unpublished reports of the number homicide offenders with schizophrenia who had committed an earlier homicide

**Region**	**Period**	**Duration of study in years**	**Total homicide offenders per 100,000**	**Number of homicides by people with schizophrenia and related psychosis**	**Number of patients with homicide recidivism N (%)**
1. Austria [33]	1975-1999	25	0.54	62	0 (0%)
2. Australia (Victoria) [34]	1993-1995	3	1.12	11	0 (0%)
3. Australia (Victoria) [21]	1997-2005	9	1.12	38	0 (0%)
4. Australia (New South Wales) [6,13]	1992-2008	17	1.78	131	1 (0.8%)
5. Barbados [35]	1978-1995	18	6.8	17	0 (0%)
6. Finland [7]	1980-1991	11	2.27	93	4 (4.3%)§
7. Germany (Hessen) [31]	1992-1996	5	0.78	17	0 (0%)
8. New Zealand [36]	1970-2000	30	1.42	74	0 (0%)
9. Netherlands [28]	2000-2006	7	1.1	24	0 (0%)
10. Nigeria (Jos) [37]	1980-1998	18	0.22	25	0 (0%)
11. Russian Federation (The Chuvash Republic) [12]	1981-2010	30	12.4	149	16† (10.7%)§
12. Saudi Arabia [26]	2008-2009	1.5	1.64	128	1 (0.8%)
13. Singapore [38]	1997-2001	15	0.63	10	0 (0%)
14. Sweden [11]	1975-2007	32	1.0	22	1 (4.5%)§

† Includes offenders who committed a first homicide prior to the onset of schizophrenia.

§ Previously published reports.

The highest proportion of people with homicide recidivism among homicide offenders with schizophrenia was found in Chuvashia, where over 30 years, 16 of 149 (10.7%) offenders with schizophrenia had committed a previous homicide (Golenkov, Large et al., [12]). Three of the 16 recidivists from Chuvashia committed the first homicide before being diagnosed with schizophrenia.

Between-study heterogeneity was high, as measured with a chi-square test (X^2^ = 46.3, df = 13, p < 0.0005). An initial estimate of the pooled proportion of homicide recidivists derived from all 14 studies by meta analysis was artificially inflated by the inclusion of notional non-zero values in the 9 studies with zero values in the numerator (needed to allow log-odds calculations). This was a particular problem because of the number of studies with small numbers of total homicides.

In order to more accurately assess the pooled estimate, the 9 studies reporting no recidivist cases were grouped into a single sample (n = 278) with the addition of a single notional recidivist case. With this correction, between-study heterogeneity remained very high (Q-value = 23.3, df = 5, P < 0.001, I-square = 79). The pooled estimate of the proportion of homicide offenders with schizophrenia who were homicide recidivists was 2.3% (95% Confidence Interval (CI) 0.07% to 7.2%).

The pooled estimate of the proportion of homicide offenders who were recidivists from the unpublished data provided by the authors of 11 known studies was considerably lower (0.06% (95% CI 0.02% to 1.8%) than the proportion of homicide offenders who were homicide recidivists from the three published reports (8.6% 95% CI 5.7%-12.9). The difference in the estimated proportions of homicide recidivists between published and unpublished data was highly significant (Q-value = 19.5, df = 1, p < 0.0005).

The 2 jurisdictions with more than one recidivist homicide (Finland and Chuvashia) had the highest and third highest total homicide rate, suggesting that jurisdictions with a high rate of homicide might have a higher rate of recidivism. Four of the 6 studies with the longest sample periods (≥18 years) reported no cases of recidivism, suggesting that study duration is not strongly associated with the proportion of recidivists among the samples of homicide offenders with schizophrenia.

## Discussion

A second homicide by a mentally ill homicide offender is not only a potentially avoidable tragedy, but has been described by Dietz as a form of ‘sensational homicide’ [39] with the potential to increase stigma experienced by people with severe mental illness and to reduce the prospects of conditional release for other mentally ill offenders [40]. While the prevention of rare events such as homicide is intrinsically difficult and the prediction of very rare events is impossible [41,42], mental health services do have some role in preventing homicides [43,44], including by focusing on co-morbid substance use of patients with an established diagnosis of psychotic illness [18,45] and by earlier treatment of first episode psychosis [46]. The prevention of homicide recidivism by people who have committed a homicide offence and are diagnosed with schizophrenia might be feasible because they are a small population with identified treatment needs.

This study suggests that in most jurisdictions, the rate of recidivist homicide offences by people with schizophrenia who have been released to community care is very low. There were no cases of homicide recidivism in New Zealand [36] or Austria [33] over periods of 30 and 25 years respectively. A low rate of recidivism was also suggested by the findings of most of the other studies from high-income countries conducted over shorter periods. Although it is not possible to draw firm conclusions about the reasons for these low rates of homicide recidivism, the most important factor in the prevention of further homicides by mentally ill offenders is likely to be the ability of forensic services to provide an adequate period of secure detention, carefully graded release to community settings and ongoing supervision of treatment after release.

This review was conducted after we published a study describing a high rate of homicide recidivism in Chuvashia [12]. Other researchers with similar findings might also be more likely to prepare a report and have it accepted for publication. Hence, there is a risk of publication bias towards reports of higher rates of recidivism, which appears to have been confirmed by our analysis of published versus unpublished data. However, the low rates of homicide recidivism in the unpublished data might also be a due to under-reporting, for example, as a result of the imperfect recollection of homicide recidivism by the primary researchers.

This study has several important limitations. First, we used unpublished data, some of which relied on the memory of the authors of the original studies. While cases of homicide recidivism are likely to be memorable, there may have been cases of which the authors were unaware. Moreover, researchers in the United Kingdom, including those associated with the Confidential Inquiry based at the University of Manchester did know of several cases, but were unable to provide data on the number of cases or the period of time in which the homicides occurred. There was also a notable lack of data from the USA, where there is a high rate of homicide and where most jurisdictions have a form of the insanity defense and secure hospital systems with a pathway to eventual community care. The absence of data from large jurisdictions and from countries with high rates of homicide, such as parts of South and Central America, and the small sample of studies we were able to locate means that our data might not be representative of all jurisdictions.

A second limitation was that in 3 of the 5 studies in which cases of homicide recidivism were reported, there was no information about whether the first offences were committed before the diagnosis of schizophrenia. Our estimate of the rates of recidivism were based on retrospective enquiry about previous homicide offences. Prospectively collected data from large populations and in jurisdictions with high rates of homicide could provide a more realistic estimate of the risk of homicide recidivism among people with schizophrenia compared to those without that disorder. A third limitation of the analysis arose from the relatively small number of studies, some of which sampled a small number of homicide offenders. Hence our estimate of recidivism among homicide offenders with schizophrenia of 2.3% should be interpreted cautiously because of the extent of between-study heterogeneity in rates and because all the studies we located reported substantially higher (3 studies) or lower (11 studies) proportions of recidivists. Our estimate could be improved on by the publication of retrospective data from a large and representative jurisdiction, such as England and Wales. Moreover, a prospective study involving several jurisdictions could provide a more clinically meaningful estimate of the probability of homicide recidivism than an estimate derived from retrospective studies.

## Conclusion

Despite significant limitations of the available data, our results suggest that repeat homicides by people with schizophrenia are rare in jurisdictions with low rates of total homicide and well developed services for the long-term treatment of homicide offenders with schizophrenia.

## Competing interests

The authors declare that they have no competing interests.

## Authors' contributions

AG, ML & ON conceived the study and drafted the paper. ML performed the searches and corresponded with the authors of the primary research. ML and ON extracted the data from published studies. ML performed the statistical analysis. All authors read and approved the final manuscript.

## Pre-publication history

The pre-publication history for this paper can be accessed here:

http://www.biomedcentral.com/1471-244X/14/46/prepub
